# Real-world treatment and survival of patients with advanced non-small cell lung Cancer: a German retrospective data analysis

**DOI:** 10.1186/s12885-020-06738-z

**Published:** 2020-03-30

**Authors:** Fränce Hardtstock, David Myers, Tracy Li, Diana Cizova, Ulf Maywald, Thomas Wilke, Frank Griesinger

**Affiliations:** 1 IPAM e.V, Alter Holzhafen 19, 23966 Wismar, Germany; 2 The Janssen Pharmaceutical Companies of Johnson & Johnson, Janssen-Cilag AB, Stockholm, Sweden; 3 The Janssen Pharmaceutical Companies of Johnson & Johnson, Janssen Global Services, Raritan, NJ USA; 4 AOK PLUS, Sternplatz 7, 01067 Dresden, Germany; 5 Universitätsklinik für Innere Medizin-Onkologie, Cancer Center Oldenburg, Pius-Hospital Universitätsmedizin Oldenburg, Georgstrasse 12, 26121 Oldenburg, Germany

**Keywords:** Non-small cell lung cancer, Advanced NSCLC, Mutation testing, Overall survival

## Abstract

**Background:**

The objective of this study was to describe the real-world treatment and overall survival (OS) of German patients with a diagnosis of advanced non-small cell lung cancer (aNSCLC), and to explore factors associated with the real-world mortality risk.

**Methods:**

This was a retrospective German claims data analysis of incident aNSCLC patients. Data were available from 01/01/2011 until 31/12/2016. Identification of eligible patients took place between 01/01/2012–31/12/2015, to allow for at least 1-year pre-index and follow-up periods. Inpatient and outpatient mutation test procedures after aNSCLC diagnosis were observed. Further, prescribed treatments and OS since first (incident) aNSCLC diagnosis and start of respective treatment lines were described both for all patients and presumed EGFR/ALK/ROS-1-positive patients. Factors associated with OS were analyzed in multivariable Cox regression analysis.

**Results:**

Overall, 1741 aNSCLC patients were observed (mean age: 66·97 years, female: 29·87%). The mutation test rate within this population was 26·31% (*n* = 458), 26·6% of these patients (*n* = 122) received a targeted treatment and were assumed to have a positive EGFR/ALK/ROS-1 test result. Most often prescribed treatments were pemetrexed monotherapy as 1 L (21·23% for all and 11·11% for mutation-positive patients) and erlotinib monotherapy as 2 L (25·83%/38·54%). Median OS since incident diagnosis was 351 days in all and 571 days in mutation-positive patients. In a multivariable Cox regression analysis, higher age, a stage IV disease, a higher number of chronic drugs in the pre-index period and no systemic therapy increased the risk of early death since first aNSCLC diagnosis. On the other hand, female gender and treatment with therapies other than chemotherapy were associated with a lower risk of early death.

**Conclusions:**

Despite the introduction of new treatments, the real-world survival prognosis for aNSCLC patients remains poor if measured based on an unselected real-world population of patients. Still, the majority of German aNSCLC patients do not receive a mutation test.

## Background

Lung cancer is the leading cause of cancer death in men and the third most frequent cause of cancer death in women worldwide, with 2·1 million new cases and 1·7 million deaths estimated for 2018 [1, 2]. In women, according to latest mortality projections, lung cancer age-standardized mortality rate will surpass breast cancer mortality rate before 2030 in many countries [3]. Non-small cell lung cancer (NSCLC) accounts for approximately 85% of all diagnosed lung cancer cases [4]. The 5-year overall survival (OS) of advanced NSCLC (aNSCLC) is about 26% in stage IIIB and 10%/1% in stage IVA/IVB patients [5].

The discovery of new molecular alterations and development of respective targeted treatments represents a major improvement over conventional chemotherapy when applied to appropriately selected patient populations [6–11]. A recent network meta-analysis on the tyrosine kinase inhibitors (TKIs) gefitinib, erlotinib and afatinib concluded in this respect that these three agents out-performed chemotherapy in terms of progression free survival (PFS), overall response rate, and disease control rate, with less clearer results with regard to OS. Similar results were shown in another meta-analysis on erlotinib only [12]. In contrast, a recent German observational study on EGFR-positive aNSCLC patients concluded that those patients who ever received a TKI during their complete therapy course, compared with those who never received a TKI, had a higher OS (median 18·4 versus 13·6 months; HR 0·53; *p* = 0·003) [13, 14].

In addition to targeted agents, recent development of immune checkpoint inhibitors such as pembrolizumab or nivolumab adds extends treatment options for aNSCLC patients, with reported median OS in clinical trials of 9·2–12·2 months (Nivolumab) and 10·4–12·7 months (Pembrolizumab, PD-L1+ patients) versus 6·0–9·4 months (Docetaxel) for chemotherapy [15–17]. On the other hand, in patients not suitable for targeted and/or immunotherapies conventional chemotherapy was still the treatment of choice until January of 2016, when pembrolizumab 1st line was approved for patients with a PD-L1 expression of > 50% [18]. OS prognosis for these patients remains poor [19–21].

Based on recent therapy developments, current European and German treatment guidelines recommend testing for mutations/mutation aberrations (EGFR, ALK, ROS-1, MET, NTRK) and PD-L1 testing, in order to identify those patients who are eligible for targeted/immune checkpoint therapies [18, 22, 23]. However, as testing procedures are associated with additional health care resource use and cost, and many questions around proper patient selection, optimal sequential/combinatorial use of agents, appropriate treatment duration etc. are still unanswered, there is an ongoing discussion about whether recent therapy developments were also associated with superior outcomes in the real-world treatment of aNSCLC patients. As most real-world data have been collected in retrospective medical chart reviews or prospective observational studies, results reported so far might be biased because of study site/patient selection procedures in these study types. That is why, the main aim of this study was to report data on mutation testing rates and treatment patterns as well as OS of aNSCLC patients in the real world, based on an unselected patient population identified in a large German claims dataset.

## Methods

### Data source

This was a retrospective claims-based data analysis of patients diagnosed with aNSCLC, using a cohort design. The study utilized data provided by the statutory German sickness fund AOK PLUS, covering routine data on health of 3.2 million people insured in Germany (regions of Saxony/Thuringia), which is more than 50% of the overall population in these states. This large claims data set includes complete records of a patient’s outpatient and inpatient diagnoses and treatments prescribed by all types of treating physicians and departments within the German healthcare system. Diagnoses were identified based on inpatient and confirmed outpatient ICD-10 codes (international classification of diseases 10th revision). Outpatient drug treatment was identified through relevant ATC-codes, and inpatient treatment was identified through relevant operational and procedure codes (OPS). The dataset covered the period 01/01/2011–31/12/2016.

### Patient selection

**Fig. 1:**
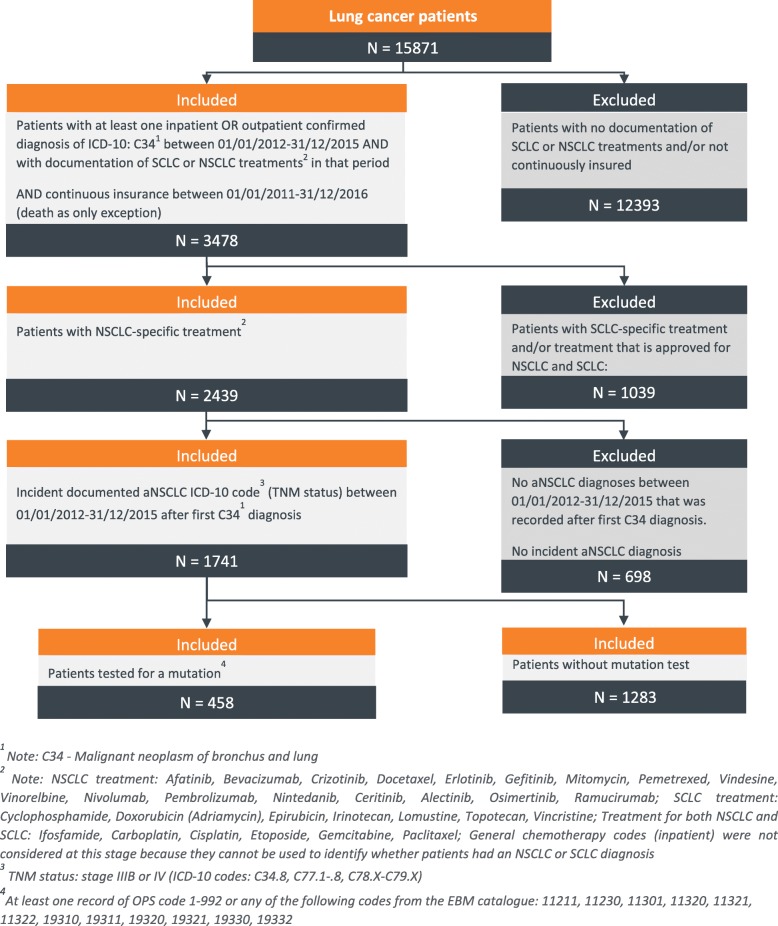
Patient attrition chart. Outlines the patient selection steps along with the patient numbers included and excluded at each step

Identification and inclusion of eligible patients took place between 01/01/2012–31/12/2015, with a baseline period of one year and a minimum follow-up period of 1 year. Patients were selected in a stepwise procedure based on their diagnoses and treatments (Fig. 1). In the first step, all patients with at least one inpatient and/or outpatient diagnosis of lung cancer (ICD-10: C34) between 01/01/2012–31/12/2015 were identified. Second, only patients continuously insured with the respective sickness fund (death being the only exception) between 01/01/2011–31/12/2016 were further analyzed. Third, as no ICD-10 code specifies type of lung cancer, NSCLC patients were identified based on documented NSCLC-specific treatments prescribed after their lung cancer diagnosis. Treatment was defined as NSCLC-specific by consulting the latest treatment guideline in Germany [24] and the official Summary of Product Characteristics (SmPCs) of the respective drugs [25]. Patients with any treatment approved for SCLC, or with a treatment approved for both SCLC and NSCLC were excluded. The complete list of SCLC and NSCLC-specific drugs (Supplementary Table 1) was additionally reviewed by an oncologist.

Fourth, out of all patients with NSCLC, patients with advanced disease stage were identified based on their tumor stage at the time of their lung cancer diagnosis. Tumor staging was evaluated with respect to the German treatment guidelines and the 8th edition of the Union for International Cancer Control’s (UICC) tumor classification, with status IIIb or higher classifying a patient as being advanced within this study. Patients with at least one confirmed inpatient or outpatient diagnosis of tumor stage IIIb or higher between 01/01/2012–31/12/2015, following/concurrent with their first observed general lung cancer diagnosis, were included (Supplementary Table 2). Fifth, only patients who did not already receive a diagnosis of advanced tumor stage in the 12 months baseline period before index date (first aNSCLC diagnosis in inclusion period) were classified as being incident aNSCLC patients.

All patients were observed for at least 12 months following their index date (or until death, whatever came first). In case of data availability, for a subset of patients, 24-month and 36-month follow-up data were analyzed.

#### Identification of mutation testing and respective results

In this study, we aimed to identify the percentage of patients with a test for an EGFR, ALK or ROS-1 mutation. Within Germany, mutation testing can be carried out in both an outpatient and an inpatient setting. Outpatient mutation tests were identified based on reimbursement codes listed in a German-wide code catalogue (Einheitlicher Bewertungsmaßstab EBM) published by the National Association of Statutory Health Insurance Physicians (Kassenärztliche Bundesvereinigung) [26]. As no unique code for above mutation tests existed in the analyzed years, we applied an extensive list of codes which were seen as proxies for a mutation test (Supplementary Table 3). Inpatient mutation tests were observed based on the documentation of a respective inpatient procedure code (OPS-code) (Supplementary Table 3). As results of the mutation tests were not available in the database, mutation-positive patients were identified under the assumption that such a mutation existed if a patient received a mutation test and, additionally, received a targeted treatment (gefitinib, erlotinib, afatinib, crizotinib) after that test (any time).

#### Identification of treatment lines

Treatments and treatment patterns were observed for all incident aNSCLC patients, and for the subgroup of mutation-positive aNSCLC patients as defined above. Prescribed treatments were identified based on respective anatomical therapeutic chemical classification (ATC) codes of agents, both in an inpatient and an outpatient setting. Generally, all prescribed outpatient treatments were available in our database, that also applied for inpatient treatments reimbursed separately outside the DRG (diagnosis related groups) system. For mostly older treatments that are not separately reimbursed in an inpatient setting, we used a “general” chemotherapy code (OPS 8–54) which documented a chemotherapy treatment but did not provide the specific agent. The first prescription of an agent after the incident aNSCLC diagnosis was defined as start of the first treatment line (1 L). Any agents prescribed on the same day or within 21 days of starting a treatment line were considered as part of a combination therapy. A start of a new treatment line was identified only when a new agent different from 1 L treatment(s) was observed after that 21 days period. Discontinuation of a treatment/treatment line was assumed if a new treatment line started or if there was a gap in drug availability of at least 45 days. In case a gap in drug availability was observed, the end of the respective gap was considered to be the end of the treatment line. In addition to that, for aNSCLC stage IIIb patients, we also reported the percentage of patients who received a radiotherapy (RT), as in these cases not necessarily a mutation test is recommended.

Identification of most frequently prescribed treatment patterns was done by observing the number of patients with a prescription of the respective monotherapy or combination therapy, within each line of therapy. Identification of the most often prescribed agents was based on all patients who received the respective agents, regardless of whether they were used as monotherapy or a combination therapy.

#### Assessment of overall survival

OS was described using Kaplan Meier methodology. The proportion of patients alive after 3/6/12 months following their index date (incident aNSCLC diagnosis), and in a subgroup analysis of patients with longer data availability, after 24 and 36 months, was reported. Furthermore, survival analysis was repeated, considering the start of 1 L/2 L/3 L treatment as index date.

Mean and median OS in days was estimated based on above assumptions. Moreover, an OS comparison between patients who (1) did not receive any systemic treatment after incident aNSCLC diagnosis, (2) received a mutation test and received a targeted therapy (at any line), (3) received a targeted therapy but never received a mutation test, (4) received an immunotherapy^1^, or (5) received chemotherapy only was done, using Log Rank tests in comparison to patient group 5 (chemotherapy only).

A multivariable Cox regression was additionally performed in order to identify the association of various independent variables such as disease stage and sociodemographic characteristics at baseline with mortality. Furthermore, type of therapy as defined above and year of incident aNSCLC diagnosis were included as independent variables.

#### Statistical analysis

Most reported data were based on descriptive statistical analyses. Baseline characteristics were compared between not-tested and mutation negative aNSCLC patients and mutation positive patients, using parametric and non-parametric tests (t-test, Chi^2^, Mann-Whitney-U). A multivariable logistic regression model was used to evaluate any potential explanatory variable for receiving a mutation test. Treatment, duration of treatment and OS were reported using descriptive statistics and applying a Kaplan Meier methodology (using Log rank tests whenever applicable). Finally, multivariable analysis of factors associated with mortality was done using Cox regression analysis, applying a backward elimination method to systematically exclude variables which were deemed insignificant based on a *p*-value of 0·1 or higher. Software used for data analysis and modeling included Microsoft Office Excel 2016 and Stata version 14·1 software (StataCorp. 2015. Stata Statistical Software: Release 14. College Station, TX: StataCorp LP).

## Results

### Baseline characteristics

Based on 15,871 identified lung cancer patients in the pre-defined inclusion period, we finally included 1741 incident aNSCLC patients, with a mean age of 67·0 years and a higher proportion of male (70·1%) than female patients (29·9%). At date of incident aNSCLC diagnosis, 32·2% of all observed patients received a diagnosis of stage IIIB NSCLC, 42·7% of stage IV and for 24·1% of patients both TNM stages had been documented at index date or within 30 days after index date. The mean Charlson Comorbidity Index (CCI) in the sample was 9·91. Within the 12-month pre-index period, patients received an average of 4·7 chronic drug prescriptions, and 76·0% experienced at least one hospitalization (Table 1).

**Table 1:** Baseline Characteristics of Study Population

Variable	All aNSCLC patients	aNSCLC patients who received a mutation test	aNSCLC patients who did not receive a mutation test	***p***-values	Mutation-positive aNSCLC patients	aNSCLC patients without a test or with negative test result	***p***-values
N	1741	458	1283	458 vs. 1283 patients	122	1619	122 vs. 1619 patients
Age at index date							
Mean	66 97	66 51	67·13	0.441	66 17	67·03	0·633
(Median | SD)	(68 |10.20)	(68 | 10.41)	(68 | 10.13)		(69 | 11.35)	(68 | 10.11)	
Gender							
Female, N (%)	520 (29·87)	176 (38·43)	344 (26·81)	< 0.001	56 (45·90)	464 (28·66)	< 0.001
TNM status at index date^1^							
IIIB^2^ (%)	32·17	31·00	32·58	0·535	26·23	32·61	0·146
IV^2^ (%)	43 71	43 45	43 80	0·896	41·80	43·85	0·660
IIIB and IV^2^ (%)	24·12	25·55	23·62	0·407	31 ·97	23·53	0 ·036
At least 1 all-cause hospitalization in baseline^2^							
% of patients	75.99	71·18	77·71	0·005	67·21	76·65	0·019
Number of chronic drugs^2,3^							
Mean	4·70	4·43	4·69	0·767	4·87	4·68	0·886
(Median | SD)	(4 | 3·91)	(4 | 4·89)	(4 | 3·92)		(4 |4·29)	(4 |3·88)	
Charlson Comorbidity Index (CCI) ^2^							
Mean	9·91	9·71	9·98	0·078	9·66	9·93	0·053
(Median | SD)	(10 | 2·75)	(9 | 2·90)	(10 | 2)		(9 | 2·62)	(10 | 2·76)	

*Legend:* Table 1 *describes baseline characteristics of observed incident aNSCLC patients as well as those of subgroups based on mutation testing and results of the mutation testing*

^*1*^*TNM stage IIIB ICD-10 codes: C34.8, C.77.0/.1/.2/.3/.4/.5/.8; TNM stage IV ICD-10 codes: C78.X-C79.X; IIIB and IV: Patients who received which had both diagnoses on the same day or within 30 days*

^*2*^*Based on 12 months baseline period*

^***3***^*Defined as at least 2 different prescriptions per ATC class in baseline period*

### Mutation testing rates

Out of 1741 observed aNSCLC patients, 458 patients (26·3%) received a mutation test at any time between first lung cancer diagnosis and end of observational time. Out of these tested patients, 122 patients (26·6%) received a targeted treatment and were thus assumed to be positively tested for EGFR, ALK or ROS-1 mutations. In total, this resulted in a rate of 7·0% mutation-positive patients, out of all identified incident aNSCLC patients (19·3% of patients with a presumed negative test result, and 73·7% of patients without testing).

Among all incident aNSCLC patients, 14·41% received a RT within the first 3 months after diagnosis (26·58% within 12 months). Among patients in stage IIIB at time of incident diagnosis (*N* = 560), corresponding numbers were 16·61% within 3 months and 29·46% within 12 months. 74·64% of aNSCLC patients within stage IIIB did not receive a mutation test. Out of those, 16·27% received a radiation therapy within 3 months (28·95% within 12 months).

Within a multivariable logistic regression model, female gender was associated with a higher probability to receive a mutation test (OR = 1·68; *p* < 0·001), whereas at least one hospitalization in the baseline period decreased that probability (OR 0·73, *p* = 0·012). TNM status, age, CCI and number of previously prescribed chronic drugs were not associated with the probability to receive a mutation test (Supplementary Table 4).

### Treatment and treatment lines

Based on a 12 month follow up, 5·3% of all observed 1741 aNSCLC patients received no systemic treatment after their incident aNSCLC diagnosis, 68·8% received a 1 L treatment only (1 L), 19·5% received 1 L and 2 L treatment (1 L + 2 L), 5·2% received three lines of treatment (1 L + 2 L + 3 L), and 1·2% received more than three lines of treatment (> 3 L; Table 2). In the 122 patients who were considered to be mutation-positive, respective numbers were 6·6% (no systemic treatment), 37·7% (1 L), 38·5% (1 L + 2 L), 13·9% (1 L + 2 L + 3 L), and 3·3% (> 3 L). The mean duration of 1 L/2 L/3 L treatment was 181.7/170.8/149.6 days in all aNSCLC patients, respectively. In the mutation-positive patient sample the respective numbers were 221.7/197.0/148.6 days.

**Table 2:** Distribution of patients with regard to systemic treatment lines

Variable		aNSCLC patients	Mutation positive aNSCLC patients
Based on a 12-month follow-up:	N^1^	1741	122
No systemic treatment	% of patients	5·34%	6·56%
1 L treatment only	% of patients	68·81%	37·70%
1 L + 2 L treatment	% of patients	19·47%	38·52%
1 L + 2 L + 3 L treatment	% of patients	5·17%	13·93%
1 L + 2 L + 3 L+ further line of treatment	% of patients	1·21%	3·28%
Based on a 24-month follow-up:	N^1^	1433	106
No systemic treatment	% of patients	4·54%	3·77%
1 L treatment only	% of patients	60·29%	16·98%
1 L + 2 L treatment	% of patients	22·40%	36·79%
1 L + 2 L + 3 L treatment	% of patients	8·79%	24·53%
1 L + 2 L + 3 L+ further line of treatment	% of patients	3·98%	17·92%
Based on a 36-month follow-up:	N^1^	1009	75
No systemic treatment	% of patients	3·87%	4·00%
1 L treatment only	% of patients	58·97%	16·00%
1 L + 2 L treatment	% of patients	22·30%	29·33%
1 L + 2 L + 3 L treatment	% of patients	9·71%	20·00%
1 L + 2 L + 3 L+ further line of treatment	% of patients	5·15%	30·67%

*Legend:* Table 2 *outlines the distribution of patients within the incident aNSCLC and mutation positive cohort with regard to treatment lines over various observational periods*

^*1*^*Note: % of patients is based on all patients, who were observable for 12/24/36 months after their incident aNSCLC diagnosis (with the only exception being death)*

Most frequently prescribed agent (regardless of monotherapy or combination regime) as 1 L treatment was pemetrexed, which was prescribed in 49·0% of aNSCLC patients and 40·2% of mutation positive aNSCLC patients. The most commonly prescribed agent in 2 L/3 L treatment was erlotinib (26·6%/40·6% as 2 L for all aNSCLC patients/mutation-positive patients, 20·2%/34·5% as 3 L).

Most frequently prescribed treatment patterns as 1 L treatment were pemetrexed monotherapy (21·2%), pemetrexed in combination with an unspecified agent (unknown agent documented during hospitalization) (7·7%), and pemetrexed and bevacizumab combination therapy (7·4%). In mutation positive patients the most commonly prescribed treatment patterns as 1 L were erlotinib (20·5%), pemetrexed (11·1%) and pemetrexed and cisplatin combination (6·8%). Most frequently prescribed treatment patterns as 2 L were erlotinib (25·8%), docetaxel (11·3%) and pemetrexed (8·0%) in all aNSCLC patients, and erlotinib (38·5%), pemetrexed (7·3%) and gefitinib (7·3%) in the subgroup of mutation positive patients (Table 3).

**Table 3:** Most frequently prescribed treatment patterns

Variable		aNSCLC patients		Mutation positive aNSCLC patients	
N		1741		122	
As 1 L treatment	Patients who started a 1 L treatment; N	1672		117	
	% patients with at least 1 prescription	21·23%	Pemetrexed	20·51%	Erlotinib
		7·72%	Pemetrexed +Unspecified	11·11%	Pemetrexed
		7·36%	Bevacizumab + Pemetrexed	6·84%	Pemetrexed + Cisplatin
		6·40%	Docetaxel	6·84%	Pemetrexed + Unspecified
		5·62%	Bevacizumab	5·98%	Bevacizumab
As 2 L treatment	Patients who started a 2 L treatment; N	635		96	
	% patients with at least 1 prescription	25·83%	Erlotinib	38·54%	Erlotinib
		11·34%	Docetaxel	7·29%	Pemetrexed
		8·03%	Pemetrexed	7·29%	Gefitinib
		5·83%	Gemcitabine	6·25%	Docetaxel + Nintedanib
		4·09%	Docetaxel + Nintedanib	6·25%	Crizotinib
As 3 L treatment	Patients who started a 3 L treatment; N	242		55	
	% patients with at least 1 prescription	20·25%	Erlotinib	34·55%	Erlotinib
		11·98%	Docetaxel	12·73%	Docetaxel
		9·92%	Pemetrexed	7·27%	Gemcitabine
		8·68%	Gemcitabine	7·27%	Vinorelbine
		5·79%	Nivolumab	5·45%	Pemetrexed
As 3 + L treatment	Patients who started a 3 + L treatment; N	90		30	
	% patients with at least 1 prescription	12·22%	Nivolumab	24·00%	Erlotinib
		10·00%	Erlotinib	16·00%	Pemetrexed
		8·89%	Nintedanib + Docetaxel	12·00%	Docetaxel
		6·67%	Pemetrexed	12·00%	Gemcitabine
		5·56%	Docetaxel	8·00%	Nivolumab

*Legend:* Table 3 *reports the most frequently prescribed treatment patterns among incident aNSCLC patients and the mutation-positive subgroup, by treatment line*

Across all treatment lines, 70·3% of patients received a chemotherapy only. A TKI-based therapy at any line was prescribed in 21·2% of the patients (might include chemotherapy and/or immunotherapy at other lines), an immunotherapy without a TKI was prescribed in 4·5% of patients (might include chemotherapy at other lines).

**Fig. 2:**
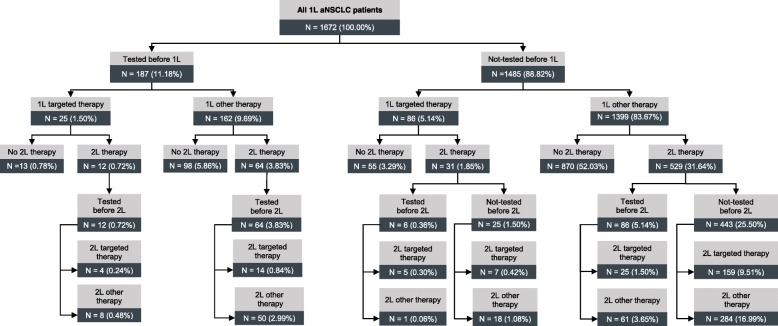
Mutation testing and observed treatment patterns over time. Describes based on all patients who received at least a 1 L treatment, distribution of mutation tests and treatment patterns over time. Treatments were divided into targeted treatments and non-targeted treatments including immunotherapy

Based on 1672 patients who started a 1 L treatment, 187 (11·2%) received at least one mutation test between incident aNSCLC diagnosis and start of 1 L treatment, and 197 (11·8%) received at least one test afterwards (Fig. 2). Among the 187 patients tested early, 25 (1·5% of all patients) received a targeted treatment. Among the 1485 patients not tested before 1 L therapy, 86 (5·1% of all patients) received a 1 L treatment with a TKI. Respective numbers for 2 L therapy are presented in Fig. 2.

Within a multivariable logistic regression model, higher age (OR = 1·01, *p* = 0·046) and a higher number of prescribed chronic drugs in the 12 months baseline period (OR = 1·03, *p* = 0·055) increased the probability to receive a chemotherapy as 1 L treatment, whereas female gender (OR = 0·51, *p* < 0·001) and stage IV disease at index date (OR = 0·60, *p* < 0·001) were found to be associated with a lower probability to receive it (Supplementary Table 5).

### Overall survival

Of the 1741 aNSCLC patients, 90·5%/73·8%/47·9% were alive after 3/6/12 months following their incident aNSCLC diagnosis. From 1388 patients who could be observed for 24 months due to data availability, 23·6% were still alive 2 years following their diagnosis, and from 958 patients who could be observed for 36 months, 14·3% were still alive 3 years following their diagnosis. From the 122 mutation-positive patients, 97·5%/91 ·8%/74·6% were alive 3/6/12 months following their incident aNSCLC diagnosis. Respective numbers for 24/36 months were 41·9%/22 5%.

**Fig. 3:**
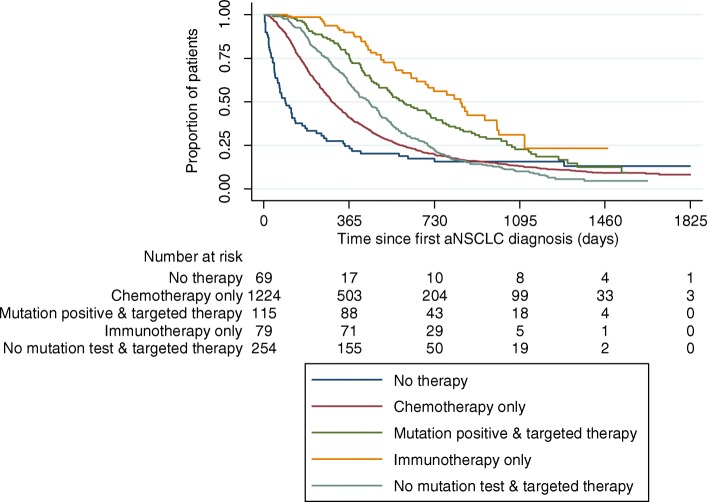
Kaplan Meier OS analysis, from date of incident aNSCLC diagnosis. Shows the Kaplan Meier survival estimates from incident aNSCLC diagnosis for the overall aNSCLC patient sample as well as for subgroups based on mutation testing and received treatments. For assignment of patients to treatments, line of therapy did not matter. Log-rank test: Chemotherapy only/No therapy: *p* = 0.001; Chemotherapy only/Mutation positive & targeted therapy: *p* < 0.001; Chemotherapy only/Immunotherapy only *p* < 0.001; Chemotherapy only/No mutation test & targeted therapy: *p* = 0.006

Kaplan Meier estimates showed that the median OS after incident diagnosis for all aNSCLC and mutation-positive patients was 351/571 days (Fig. 3). Median OS of patients from date of start of 1 L/2 L/3 L treatment was 301/194/174 days respectively. Median OS of all aNSCLC patients/mutation positive patients did not change over time, with 319 days for patients with first aNSCLC diagnosis in 2015, and 332/392/356 days for those first diagnosed in 2012/2013/2014.

**Fig. 4:**
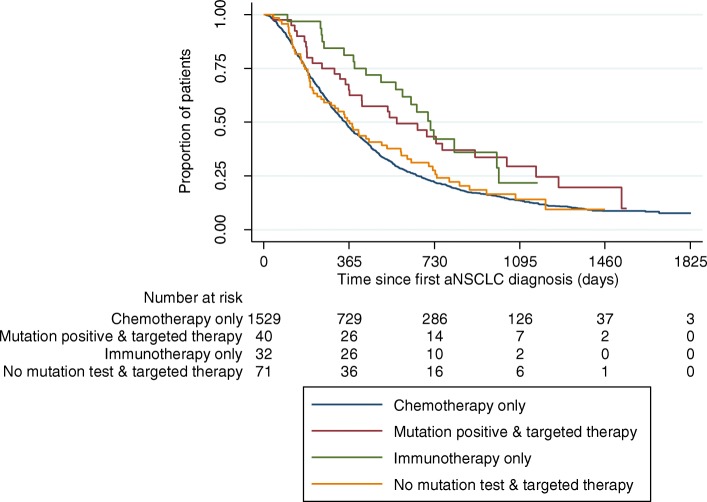
Kaplan Meier OS analysis, from date of incident aNSCLC diagnosis by 1 L treatment type. Shows the Kaplan Meier survival estimates from incident aNSCLC diagnosis for the overall aNSCLC patient sample as well as for subgroups based on mutation testing and received 1 L treatments. Log-rank test: Chemotherapy/Mutation positive & targeted therapy: *p* = 0.011; Chemotherapy/Immunotherapy only *p* = 0.002; Chemotherapy/No mutation test & targeted therapy: *p* = 0.570

In a comparison of different treatment patterns across all lines, patients who received at least once an immunotherapy or a TKI combined with a mutation test had the highest OS since incident aNSCLC diagnosis (*p* < 0·001 in comparison to chemotherapy group). OS of patients having received a TKI without a mutation test was lower but still significantly higher than in the chemotherapy only group (*p* = 0·006). Lowest OS was observed for patients who did not receive any therapy (*p* = 0·001 in comparison to chemotherapy only group) (Fig. 3). If respective treatments were compared with regard to OS since start of 1 L therapy, and only type of 1 L therapy was taken into account, highest OS was observed for immunotherapy patients (*p* = 0·002 in comparison to chemotherapy), followed by mutation-positive patients who received a TKI (*p* = 0 ·011 in comparison to chemotherapy) (Fig. 4). Survival of patients without a test but receiving a TKI and patients who received a chemotherapy was not statistically different from each other (*p* = 0·570).

**Fig. 5:**
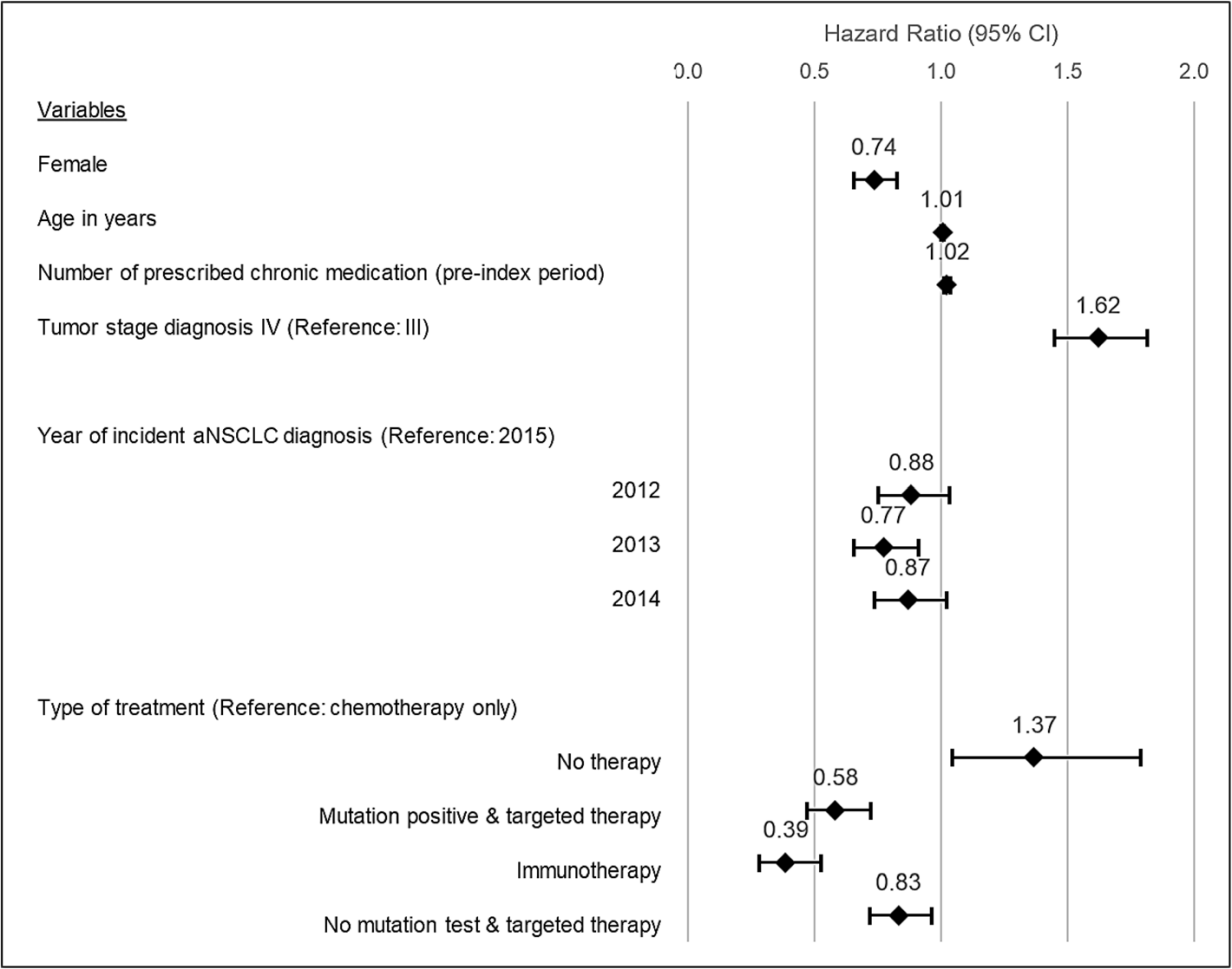
Multivariate Cox regression analysis of factors associated with early death, since date of incident aNSCLC diagnosis. Shows the results of a multivariable Cox regression analysis exploring predictors of early death. Variables initially included but excluded due to their insignificance were positive mutation status and hospitalizations in pre-index period

In a multivariable Cox regression analysis (Fig. 5) using time to death as dependent variable, higher age (HR = 1·01, *p* = 0·013), a stage IV disease (HR = 1·62, *p* < 0·001), a higher number of chronic drugs in the pre-index period (HR = 1·03, *p* < 0.001) and no therapy (HR = 1·34, *p* = 0·031) increased the risk of early death since incident aNSCLC diagnosis. Conversely, female gender (HR = 0·73, *p* < 0·001), an incident diagnosis in 2013 (HR = 0·77 in comparison to 2015, *p* = 0·002) and treatment with therapies other than conventional chemotherapy (mutation positive & targeted therapy: HR = 0·59, *p* < 0·001; immunotherapy only: HR = 0·36 *p* < 0·001; no mutation test & targeted therapy: HR = 0·85; *p* = 0 026) were associated with a lower risk of early death. Positive mutation status, number of hospitalizations in the pre-index period were not associated with mortality risk and, consequently, excluded from the final regression models.

## Discussion

The main aim of this study was to analyze the real-world treatment of aNSCLC patients and their OS in Germany. The main strength of this analysis is the unselected nature of the dataset which included all aNSCLC patients and all treating physicians irrespective of their type and willingness to participate in a clinical or observational study, with a resulting high external validity of our results. Moreover, our study completely covered both the inpatient and outpatient treatment of patients and, consequently, all sectors of the German healthcare sector. As we included all patients meeting inclusion criteria, our study was also not affected by any selection bias that would lead to an observation of an above-average treated patient sample only, as it can be expected to be the case in most of conducted observational studies/registries. However, we acknowledge that specifically our treatment-related inclusion criteria might have led to an own selection bias that excluded untreated patients as well as patients receiving treatments approved for SCLC and NSCLC from further observation. Moreover, a substantial proportion of patients was already excluded due to non-continuous insurance. We cannot draw conclusions with regard to these excluded patients, as we aimed to observe aNSCLC patients only. As the characteristics of our sample are well in line with previous clinical and observational studies with respect to age, gender distribution and percentage of IIIb/IV patients [27–29], we nevertheless interpret our results to be generalizable with regard to treatment and OS of aNSCLC patients in Germany.

With the exception of aNSCLC stage IIIb patients who receive a potentially curative RT, German diagnosis and treatment guidelines recommend testing for mutations for every diagnosed aNSCLC patient before start of a 1 L treatment [24]. However, in our sample, only 26·3% of the observed patients were tested at all, and only 11·2% received a test between incident aNSCLC diagnosis and start of 1 L therapy. Only the minority of them were IIIb patients who received a RT. Testing frequency did not increase as the developments and understanding in oncologic mutations progressed over the years, as among patients diagnosed in 2015 only 24·9% of patients were tested. Our result might have been influenced by the fact that we used proxies for identification of mutation tests, by applying a wide range of outpatient genetic testing codes for identification of mutation tests (Supplementary Table 3). That is why we validated our proxy codes by a subgroup analysis dealing with patients diagnosed with aNSCLC since 01/01/2016 only. From that date onwards, a specific code for mutation tests had been introduced in Germany. In this analysis, only 19·9% of patients were tested based on the new coding system. So, our above proxies seem to be reliable, and might due to their nature even overestimate the proportion of tested patients.

96·7% of observed patients were hospitalized since incident aNSCLC diagnosis at least once, 59·6% of observed patients were hospitalized at date of their incident aNSCLC diagnosis. Mutation tests done during inpatient stays might not have been documented properly in our study even if the specific codes are available, as they are not separately reimbursed within the German diagnosis related groups (DRG) system. That would have led to an underestimation of the mutation test rate. However, as German hospitals currently do not get any reimbursement for those tests on top of the DRGs, we do not expect that a substantial percentage of patients was tested during their inpatient stays.

Inclusion of mutation testing into the real-world treatment of aNSCLC patients has been widely discussed in scientific literature [30–35]. For example, a study using US claims data reported that 18% of newly diagnosed patients with metastatic lung cancer had claims for EGFR testing within 6 months following diagnosis [30]. In qualitative interviews with oncologists treating NSCLC patients in the US, only a minority reported testing every patient regardless of stage, histology, age and smoking status [32]. On the other hand, an example from the Netherlands showed that implementation of guideline recommendations into clinical practice in four hospitals increased the mutation testing rate from 20 ·8% to 74·4% within 6 years [36].

Almost 95% of our sample of German aNSCLC patients received a systemic treatment. This is, compared to previous literature, relatively high. Sacher et al. reported that only 24% of patients from a large scale data registry in Canada received 1 L chemotherapy and only 31% of those patients received 2 L therapy with more patients receiving treatment over time [37]. David et al. utilizing the National Cancer Data Base (NCDB) in the US reported that 22·3% of stage IIIB and 25·9% of stage IV NSCLC patients received no treatment, and 13·9% and 21·6% received radiation with no systemic chemotherapy, respectively. Our percentage of patients who received a systemic treatment might overestimate the true quota in this respect, as only lung cancer patients who received a NSCLC treatment (not necessarily since first aNSCLC diagnosis, but since first lung cancer diagnosis) were observed in our study.

Even if almost all of our observed patients received a systemic treatment, the majority of them only received one systemic treatment line within the first year following incident aNSCLC diagnosis (68·8%; mutation-positive: 37·7% of all patients). Albeit difficult to observe from claims data records, the most frequent treatment patterns used in 1 L treatment were pemetrexed and bevacizumab, related combinations (41·93%) and docetaxel (6 ·40%). This is in line with the treatment guidelines, which recommend 1 L treatment with pemetrexed or bevacizumab platinum based combinations in patients without mutation and non-squamous NSCLC [24, 38]. Pemetrexed combinations as frequent 1 L treatments were also observed in published literature. Bittoni et al. reported 12% of patients within the US SEER-Medicare database receiving a pemetrexed and carboplatin combination from 2007 through mid-2013 [39], and Sztankay et al. reported 40·5% of patients within three Austrian medical centres receiving 1 L pemetrexed and cisplatin, and 33·3% receiving 1 L pemetrexed and carboplatin [40].

In mutation-positive patients, however, a substantial percentage of > 20% of patients received a pemetrexed-based 1 L treatment, which is not according to guidelines. This might be related to the fact that more than half of observed mutation tests were done after start of 1 L therapy. On the other hand, some positively tested patients might still receive non-targeted therapies in clinical practice, which is clearly disadvantageous to patients.

We report a general median survival of 11·5 months in our aNSCLC sample. Mutation-positive patients, based on our proxy definition, had a better median OS of 18·8 months in comparison to the 11·1 months in the not tested/negatively tested patients. Our median OS is well in line with what has been reported in previous observational studies. A US study addressing a community oncology setting reported a median OS of 9·7 months for an aNSCLC patient sample without known EGFR mutations [41]. A European study could identify a median OS of 10·3 months for aNSCLC patients treated with a 1 L platinum-based chemotherapy [42]. A multinational observational study observed a median OS from first-line therapy initiation of 10·0 (Japan) to 26·7 (Taiwan) months for those tested, and 7·6 (Australia/Brazil) to 19·3 (Taiwan) months for those not tested. Our higher median OS for those patients with a presumed positive testing result is also in line with numbers from previous studies, which reported an OS of up to 39·6 months [14, 36, 43–46]. Additionally, we are very much in line with a previous German observational study which reported a median OS of 18·1–18·4 months for mutation-positive patients who received a TKI during their treatment course [14].

Our multivariable analysis shows that both immunotherapy treatments and TKI treatments are associated with a better OS. However, this result might be strongly influenced by unobserved differences in characteristics of compared patients, and can thus not be generalized to all aNSCLC patients. On the other hand, as many new treatments became available to patients in recent years, we expected an improvement of median OS for the overall aNSCLC population diagnosed in recent years. However, we could not confirm this. OS of patients diagnosed in later years 2014–2015 was not better than that of patients diagnosed in 2012–2013. In association with the low testing rates this shows that the real-world treatment of aNSCLC patients’ needs to be urgently improved.

### Limitations

We acknowledge some limitations which are mostly related to the nature of the analyzed data. Firstly, we used a retrospective anonymous claims data set which combines the advantages of full data coverage over different sectors of healthcare (inpatient and outpatient treatment) and absence of any study site/patient selection bias due to the study itself with the disadvantage of lack of clinical details as mainly data used for reimbursement purposes are available in such a dataset. Nevertheless, claims data are a often used data source for observational research, and have frequently been used in the area of lung cancer as well [30, 39]. In addition to that, usage of a regional claims database as the used AOK PLUS database might imply a bias by itself. However, due to the German legal framework, health service reimbursement rules are identical across Germany, so that no bias could result from using a database from one database. There remains the risk of a regional bias, as AOK PLUS insures persons in two German states only. We cannot rule out this bias risk completely, but previous studies in non-cancer diseases done with AOK PLUS data as well that there are no major regional differences in health care structures and services between Saxon/Thuringia and other German states [47].

Secondly, as already noted above, our method to identify mutation tests might have led to both an under- and overestimation of testing rates. Thirdly, our method of identifying the index dates of the patients in the included sample might have led to the overestimation of OS. The index date was identified as the first inpatient or outpatient incident aNSCLC diagnosis. Whereas the exact dates of inpatient data were available, for outpatient diagnoses only the respective quarter of the year was available. Here, we used the first day in a quarter as index date. In our dataset, 56·3% of all aNSCLC patients (981 out of 1741 patients) were included based on an inpatient diagnosis as index date. For the remaining 760 patients (43·7%), above mentioned overestimation of up to three months might have occurred.

Fourthly, mutation-positive patients were identified based on whether they received a targeted treatment any time following an observed mutation test. In case a patient received a mutation test with a negative result, but still received a targeted treatment at any time after that test, we wrongly assumed that this patient was tested positively. Moreover, it should be noted that according to German treatment guidelines, Erlotinib can also be prescribed as 2 L+ treatment in mutation negative NSCLC patients in case other therapy options are not suitable, which might have led to an overestimation of the percentage of mutation positive patients in the analysis. Furthermore, taking into account targeted treatment as an inclusion criterion for the sample of mutation positive aNSCLC patients, reporting of the most often prescribed agents in 1 L, 2 L or 3 L may have been biased, even if all observed treatments within the selected patient group have been reported.

Fifth, as aNSCLC patients are likely to be hospitalized, inpatient chemotherapy treatment had to be considered throughout the analysis. However, for older chemotherapy agents not reimbursed separately by German payors but as part of DRGs, a chemotherapy was identifiable via a specific operational procedure code, but not the type of agent. Thus, we assumed that any unspecified chemotherapy treatment during a hospitalization was the same as the one prior to the inpatient stay, which could have led to an underestimation of lines of therapy patients received if many inpatient hospitalizations were associated with a change of chemotherapy.

Sixth, treatments administered within clinical studies might not have been recorded in our claims dataset as study medication is paid by the sponsors of these studies. Moreover, the duration of treatment lines was likely to be overestimated as the end and not the beginning of a time gap (45 days) in treatment prescriptions was assumed to be the end of the treatment line. Finally, identification of treatment lines in this analysis did not take into account maintenance therapies, which might have led to a higher than actual number of treatment lines being reported.

## Conclusions

Median OS of German aNSCLC patients in the real world is low, but in line with other countries. Patients with a positive EGFR/ALK/ROS-1 mutation test in association with a targeted treatment do have a better OS than those not receiving a targeted treatment and/or those who did not receive a mutation test.

Despite the introduction of new treatments in aNSCLC, the real-world survival prognosis for patients in advanced stages of NSCLC did not change in recent years. Mutation testing for aNSCLC patients is still not a common practice, despite clear guideline recommendations and availability of targeted treatments. Therefore, there is an urgent need to improve the real-world treatment of these patients.

## Supplementary information

**Additional file 1: Table S1.** Treatments approved for NSCLC and/or SCLC

**Additional file 2: Table S2.** Advanced tumor stage classification descriptions and respective ICD-10 codes

**Additional file 3: Table S3.** Mutation test codes

**Additional file 4: Table S4.** Logistic regression model for mutation testing

**Additional file 5: Table S5.** Logistic regression model for type of 1L treatment

## Data Availability

The data used in this study are abstracted from individual patient records and are not publicly available.

## Abbreviations

ALK: Anaplastic lymphoma kinase
aNSCLC: Advanced non-small cell lung cancer
ATC: Anatomical therapeutic chemical classification
CCI: Charlson Comorbidity Index
DRG: Diagnosis related groups
EGFR: Epidermal growth factor receptor
NCDB: National Cancer Data Base
NSCLC: Non-small cell lung cancer (NSCLC)
OPS: Operational and procedure codes
OR: Odds Ratio
OS: Overall survival
ROS-1: Proto-oncogene receptor tyrosine kinase
RT: Radiotherapy
SmPCs: Summary of Product Characteristics
TKI: Tyrosine kinase inhibitor
UICC: Union for International Cancer Control’s
