# Supplementary material for: Real-world treatment and survival of patients with advanced non-small cell lung Cancer: a German retrospective data analysis
Source: BMC Cancer. 2020 Mar 30;20:260. doi: 10.1186/s12885-020-06738-z (PMC7106673; doi:10.1186/s12885-020-06738-z)
Supplement: Supplementary file 1 — Additional file 1: Table S1. Treatments approved for NSCLC and/or SCLC [file 12885_2020_6738_MOESM1_ESM.docx]

Supplementary table 1: Treatments approved for NSCLC and/or SCLC

| **Agent** | **Outpatient medication:**  **ATC code** | **Inpatient medication:**  **OPS code** | **Specification SCLC/NSCLC** |
| --- | --- | --- | --- |
| Afatinib | L01XE13 |  | NSCLC |
| Bevacizumab | L01XC07 | 6-002.9 | NSCLC |
| Crizotinib | L01XE16 | 6-006.c | NSCLC |
| Docetaxel | L01CD02 | 6-002.h | NSCLC |
| Erlotinib | L01XE03 |  | NSCLC |
| Gefitinib | L01XE02 |  | NSCLC |
| Mitomycin | L01DC03 |  | NSCLC |
| Pemetrexed | L01BA04 | 6-001.c | NSCLC |
| Vindesine | L01CA03 |  | NSCLC |
| Vinorelbine | L01CA04 |  | NSCLC |
| Nivolumab | L01XC17 | 6-008.m | NSCLC |
| Pembrolizumab | L01XC18 | 6-009.3 | NSCLC |
| Nintedanib | L01XE31 |  | NSCLC |
| Ceritinib | L01XE28 | 6-008.a | NSCLC |
| Alectinib | L01XE36 |  | NSCLC |
| Osimertinib | L01XE35 |  | NSCLC |
| Ramucirumab | L01XC21 | 6-007.m | NSCLC |
| Ifosfamide | L01AA06 |  | Both |
| Carboplatin | L01XA02 |  | Both |
| Cisplatin | L01XA01 |  | Both |
| Etoposide | L01CB01 |  | Both |
| Gemcitabine | L01BC05 | 6-001.1 | Both |
| Paclitaxel | L01CD01 | 6-001.f/6-005.d | Both |
| Cyclophosphamide | L01AA01 |  | SCLC |
| Doxorubicin (Adriamycin) | L01DB01 | 6-001.b/6-002.8 | SCLC |
| Epirubicin | L01DB03 |  | SCLC |
| Irinotecan | L01XX19 | 6-001.3 | SCLC |
| Lomustine | L01AD02 |  | SCLC |
| Topotecan | L01XX17 | 6-002.4 | SCLC |
| Vincristine | L01CA02 |  | SCLC |
| Unspecified inpatient chemotherapy |  | 8-542/8-543/8-8-544 |  |
