# Supplementary material for: Real-world treatment and survival of patients with advanced non-small cell lung Cancer: a German retrospective data analysis
Source: BMC Cancer. 2020 Mar 30;20:260. doi: 10.1186/s12885-020-06738-z (PMC7106673; doi:10.1186/s12885-020-06738-z)
Supplement: Supplementary file 2 — Additional file 2: Table S2. Advanced tumor stage classification descriptions and respective ICD-10 codes [file 12885_2020_6738_MOESM2_ESM.docx]

Supplementary table 2: Advanced tumor stage classification descriptions and respective ICD-10 codes

| **Tumor stage classification** | **ICD-10 codes** | **Description** |
| --- | --- | --- |
| **Stage IIIB** |  |  |
| **T4,N2,M0** | C34.8 | Malignant neoplasm of overlapping sites of bronchus and lung |
|  | C77.1 | Secondary and unspecified malignant neoplasm of intrathoracic lymph nodes |
| **Any T+N3+M0** | C77.0 | Secondary and unspecified malignant neoplasm of lymph nodes of head, face and neck |
|  | C77.2 | Secondary and unspecified malignant neoplasm of intra-abdominal lymph nodes |
|  | C77.3 | Secondary and unspecified malignant neoplasm of axilla and upper limb lymph nodes |
|  | C77.4 | Secondary and unspecified malignant neoplasm of inguinal and lower limb lymph nodes |
|  | C77.5 | Secondary and unspecified malignant neoplasm of intrapelvic lymph nodes |
|  | C77.8 | Secondary and unspecified malignant neoplasm of lymph nodes of multiple regions |
| **Stage IV** |  |  |
| **Any T, any N, M1** | C78.X | Secondary malignant neoplasm of respiratory and digestive organs |
|  | C79.X | Secondary malignant neoplasm of other and unspecified sites |
