# Supplementary material for: Real-world treatment and survival of patients with advanced non-small cell lung Cancer: a German retrospective data analysis
Source: BMC Cancer. 2020 Mar 30;20:260. doi: 10.1186/s12885-020-06738-z (PMC7106673; doi:10.1186/s12885-020-06738-z)
Supplement: Supplementary file 3 — Additional file 3: Table S3. Mutation test codes [file 12885_2020_6738_MOESM3_ESM.docx]

Supplementary table 3: Mutation test codes

| **Code** | **Description** |
| --- | --- |
| **INPATIENT** | |
| **OPS code**  1-992 | Performing gene mutation analysis and gene expression analysis on solid malignant neoplasms |
| **OUTPATIENT^1^** | |
| **Test code** | **Description** |
| 11211 | Basic genetic testing benefits for insured persons from the beginning of the 6th until the completion 59th year of life |
| 11230 | Scientific-based human genetic assessment |
| 11301 | Basic fee for human genetic in-vitro diagnostics at the time of sample submission |
| 11320 | Detection or exclusion of a disease-relevant or disease-causing genomic mutation by hybridization with a mutation sequence-specific probe |
| 11321 | Detection or exclusion of a disease-relevant or disease-causing genomic mutation by means of sequence-specific and non-carrier-bound nucleic acid amplification |
| 11322 | Detection or exclusion of a disease-relevant or disease-causing genomic mutation by sequencing of human DNA by the Sanger chain termination method |
| 19310 | Histological or cytological examination of a material |
| 19311 | Cytological examination of a material |
| 19320 | Histological or cytological examination of a material using a special immunochemical procedure |
| 19321 | Immunohistochemical and / or immunocytochemical detection of receptors |
| 19330 | Cytological examination of a material with DNA determination |
| 19332 | Histological topography-specific determination (s) and identification (s) of the cell or tissue structure (s) to be examined on morphological examination material in connection with Fee Charges 11320, 11321 and 11322 |

Note: The validity of this proxy was tested by comparing the recorded frequency of testing based on old codes in the inclusion period, with the recorded frequency in 2016/2017 based on old and new (more accurate) codes released in the second half of 2016.^1^Outpatient codes for mutation testing from the EBM catalogue were only included if they were reported at least once in the entire patient sample
