# Supplementary material for: Real-world treatment and survival of patients with advanced non-small cell lung Cancer: a German retrospective data analysis
Source: BMC Cancer. 2020 Mar 30;20:260. doi: 10.1186/s12885-020-06738-z (PMC7106673; doi:10.1186/s12885-020-06738-z)
Supplement: Supplementary file 4 — Additional file 4: Table S4. Logistic regression model for mutation testing [file 12885_2020_6738_MOESM4_ESM.docx]

Supplementary table 4: Logistic regression model for mutation testing

| N = 458 patients were tested | | | | | |
| --- | --- | --- | --- | --- | --- |
| **Pseudo R^2^ = 0.0136** | **Dependent variable: Receiving a mutation test**  **(Reference = not receiving a mutation test)** | | | | |
| **Independent variable** |  | **N** | **Odds ratio** | **p-value** | **95% Confidence interval** |
| **Gender** | Males | 1221 | Reference | - | - |
|  | Females | 520 | 1·68 | < 0·001 | 1·34 – 2. ·10 |
| **At least 1 all-cause hospitalization in baseline period** | No | 560 | Reference | - | - |
|  | Yes | 1181 | 0·73 | 0·012 | 0·57 – 0·93 |

Backward elimination of the following variables because of insignificance (defined as p>0·1): TNM status IV at index date, Age at index date, Number of chronic drugs prescribed in baseline period^[[1]](#footnote-1)^, CCI based on baseline period

1. Defined as at least 2 different prescriptions per ATC class [↑](#footnote-ref-1)
