# Supplementary material for: Real-world treatment and survival of patients with advanced non-small cell lung Cancer: a German retrospective data analysis
Source: BMC Cancer. 2020 Mar 30;20:260. doi: 10.1186/s12885-020-06738-z (PMC7106673; doi:10.1186/s12885-020-06738-z)
Supplement: Supplementary file 5 — Additional file 5: Table S5. Logistic regression model for type of 1L treatment [file 12885_2020_6738_MOESM5_ESM.docx]

Supplementary table 5: Logistic regression model for type of 1L treatment

| N = 1672 patients who received 1L treatment (1194/478 with chemotherapy/other 1L treatment) | | | | | |
| --- | --- | --- | --- | --- | --- |
| **Pseudo R^2^ = 0.0322** | **Dependent variable: Receiving chemotherapy as 1L treatment (Reference = other treatment)** | | | | |
| **Independent variable** |  | **N** | **Odds ratio** | **p value** | **95% Confidence interval** |
| **Age** |  |  | 1·01 | 0·046 | 1·00 – 1·02 |
| **Gender** | Males | 1173 | Reference | - | - |
|  | Females | 499 | 0·51 | < 0·001 | 0·41 – 0·64 |
| **TNM status at start of**  **1L treatment** | IIIb | 593 | Reference | - | - |
|  | IV | 1079 | 0·60 | < 0·001 | 0·48 – 0·77 |
| **Number of prescribed chronic drugs in the 12 months before start of 1L** | | | 1·03 | 0·055 | 1·00 – 1·06 |

Backward elimination of the following variables because of insignificance (defined as p>0·1): Age, CCI based on 12 months before start of 1L, Number of all-cause hospitalizations in the 12 months before start of 1L
